# Effectiveness of cognitive rehabilitation on mild cognitive impairment using teleneuropsychology

**DOI:** 10.1590/1980-5764-DN-2022-0079

**Published:** 2023-07-24

**Authors:** Carlos Martínez Canyazo, Greta Keller, Belen Helou, Micaela Arruabarrena, Nicolas Corvalán, Agostina Carello, Paula Harris, Monica Feldman, Rodrigo Fernández, Ismael Luis Calandri, María Eugenia Martin, Ricardo Francisco Allegri, Lucía Crivelli

**Affiliations:** 1 Hospital Fleni, Departamento de Neurología Cognitiva, Buenos Aires, Argentina.; 2 Hospital Fleni, Servicio de Neuroinmunología, Buenos Aires, Argentina.; 3 Universidad de Buenos Aires, Instituto de Psicología, Biología Molecular y Neurociencias, CONICET, Buenos Aires, Argentina.; 4 Universidad de la Costa, Departamento de Ciencias de la Salud, Barranquilla, Colombia.; 5 Instituto de Neurociencias, Fleni-CONICET, Buenos Aires, Argentina.

**Keywords:** Neuropsychology, COVID-19, Cognitive Dysfunction, Memory, Neuropsicologia, COVID-19, Disfunção cognitiva, Memória

## Abstract

The COVID-19 pandemic has affected the continuity of cognitive rehabilitation worldwide. However, the use of teleneuropsychology to provide cognitive rehabilitation has contributed significantly to the continuity of the treatment.

**Objectives:**

To measure the effects of cognitive telerehabilitation on cognition, neuropsychiatric symptoms, and memory strategies in a cohort of patients with mild cognitive impairment.

**Methods:**

A sample of 60 patients with mild cognitive impairment according to Petersen’s criteria was randomly divided into two groups: 30 treatment cases and 30 controls (waiting list group). Subjects were matched by age, sex, and Montreal Cognitive Assessment. The treatment group received ten cognitive telerehabilitation sessions of 45 minutes duration once a week. Pre-treatment (week 0) and post-treatment (week 10) measures were assessed for both groups. Different linear mixed models were estimated to test treatment effect (cognitive telerehabilitation vs. controls) on each outcome of interest over time (pre/post-intervention).

**Results:**

A significant group (control/treatment) x time (pre/post) interaction revealed that the treatment group at week 10 had better scores in cognitive variables: memory (RAVLT learning trials p=0.030; RAVLT delayed recall p=0.029), phonological fluency (p=0.001), activities of daily living (FAQ p=0.001), satisfaction with memory performance (MMQ satisfaction p=0.004) and use of memory strategies (MMQ strategy p=0.000), as well as, and a significant reduction of affective symptomatology: depression (GDS p=0.000), neuropsychiatric symptoms (NPI-Q p=0.045), forgetfulness (EDO-10 p=0.000), and stress (DAS stress p=0.000).

**Conclusions:**

Our study suggests that CTR is an effective intervention.

## RESUMO

A pandemia do COVID-19 afetou a continuidade da reabilitação cognitiva em todo o mundo. No entanto, o uso de tele neuropsicologia para a reabilitação cognitiva tem contribuído significativamente para a continuidade do tratamento.

**Objetivos:**

Medir os efeitos da tele reabilitação cognitiva na cognição, nos sintomas neuropsiquiátricos e nas estratégias de memória em uma coorte de pacientes com comprometimento cognitivo leve.

**Métodos:**

Uma amostra de 60 pacientes com comprometimento cognitivo leve de acordo com os critérios de Petersen foi dividida aleatoriamente em dois grupos: 30 casos de tratamento e 30 controles (grupo de lista de espera). Os assuntos foram pareados por idade, sexo e Avaliação Cognitiva de Montreal. O grupo de tratamento recebeu dez sessões de tele reabilitação cognitiva de 45 minutos de duração uma vez por semana. As medidas pré-tratamento (semana 0) e pós-tratamento (semana 10) foram avaliadas para ambos os grupos. Diferentes modelos lineares mistos foram estimados para testar o efeito do tratamento (tele reabilitação cognitiva vs. controles) em cada desfecho de interesse ao longo do tempo (pré-/pós-intervenção).

**Resultados:**

Uma interação significativa grupo (controle/tratamento) x tempo (pré/pós) revelou que o grupo de tratamento teve melhores pontuações em variáveis cognitivas na semana 10: memória (ensaios de aprendizagem RAVLT p = 0,030; RAVLT recordação tardia p=0,029), fluência fonológica (p=0,001), atividades da vida diária (FAQ p=0,001), satisfação com o desempenho da memória (satisfação MMQ p=0,004) e uso de estratégias de memória (estratégia MMQ p=0,000), bem como uma significativa redução da sintomatologia afetiva: depressão (GDS p=0,000), sintomas neuropsiquiátricos (NPI-Q p=0,045), esquecimento (EDO-10 p=0,000) e estresse (DAS estresse p=0,000).

**Conclusões:**

Nosso estudo sugere que a CTR é uma intervenção eficaz.

## INTRODUCTION

From March 20, 2020, to December 2021, Argentina applied the Mandatory Preventive Social Isolation (ASPO) due to the coronavirus pandemic (COVID-19). This process has undergone different phases with varying intensity and strictness in the restrictions, although with the constant recommendation of Mandatory Preventive Social Distancing (DSPO) for both general and vulnerable populations.

Older adults, the population with the highest prevalence of chronic pathologies (diabetes, heart disease, obesity, metabolic syndromes, etc.), are the most vulnerable to the virus^1^. For this reason, it is still suggested that they take proper precautions or even avoid risks such as traveling by bus or going to crowded places like hospitals. In response to the global impact of the COVID-19 pandemic, cognitive telerehabilitation (CTR) has emerged as a promising alternative model to traditional in-person clinical visits^2^.

Traditional face-to-face cognitive rehabilitation (CR) has proven useful in reducing or stabilizing cognitive decline in different neurological conditions. Multiple studies and systematic reviews have shown that traditional CR has positive effects on mild cognitive impairment (MCI) patients, improving their cognitive abilities and quality of life^3–8^. Still, traditional face-to-face treatment may be very expensive and not accessible due to several barriers, such as distance, patient’s physical or financial difficulties, or lack of caregivers to accompany the patient to the appointment^9^.

With the advent of technology and the consequent removal of imposed temporal and spatial constraints, CTR has recently gained popularity in cognitive intervention for patients with cognitive impairment^10^. CTR includes any home-based cognitive intervention in which digital technology (mobile phones, video, sensors, internet platforms, etc) is used to provide neuropsychological services to patients^11^. The asynchronous modality of CTR allows the patient to plan the time devoted to rehabilitation with a degree of freedom, allowing the therapist to prescribe high-intensity training for the long term for a wide range of older individuals^12,13^.

As demonstrated by Cotelli et al.^14^ in their comprehensive review, CTR can be as successful as in-person therapies. The included studies demonstrated that using feedback from the therapist has a favorable impact on performance, engagement, and motivation, and is a critical component for a successful CTR implementation. In this direction, a home-based telerehabilitation asynchronous program tested in patients with stroke^13^ found good participant compliance and was effective in providing education and secondary stroke prevention to participants.

The high prevalence of MCI worldwide^15^ and in our population^16^ made access to CTR a priority. Promoting active and healthy aging has become crucial to preserve physical, social, and cognitive functioning and increase cognitive reserve. While these care measures are paramount, the pandemic undermined the continuity of traditional face-to-face CR. That is why the use of CTR has been boosted by the rise of the COVID-19 worldwide and in our region as an alternative.

The COVID-19 pandemic-related isolation measures have compromised in-person access to health care. However, it has opened the door to telehealth and CTR providing an alternate form of access to the healthcare system for patients. CTR is a way to allow them to continue their treatments and avoid restrictions from the social distancing measures. This is the reason it’s fundamental to assess and register the efficacy of this new modality of treatment delivery. The CTR could contribute to broader access to the health system in territorial terms and substantially improve the quality of life of people who have difficulties accessing treatment due to issues related to social distancing. This is the first study to evaluate the effects of CTR in Latin America.

## METHODS

### Study design and participants

In this study, we investigated CTR cognitive and neuropsychiatric effects. It is a non-blinded randomized clinical study. Sixty patients with MCI, attending CR at the Cognitive Neurology Service of Fleni (Buenos Aires, Argentina) were randomly selected according to Petersen’s criteria^17,18^. Both patients and their families received information on the patients’ diagnosis. Inclusion criteria were people with MCI and age >60 years. Exclusion criteria were alcohol or drug abuse, visual or auditory deficits that hinder correct cognitive ability, and history of major psychiatric disorders. Before the inclusion in the treatment groups, all participants signed an informed consent form approved by the institution’s Ethics and Research Committee.

Subsequently, participants were randomly divided into two groups: 30 treatment cases and 30 controls (waiting list group).

The CTR treatment was performed by a team of neuropsychologists with extensive experience using the AgeWise program^19^. The AgeWise program is a multicomponent intervention. It includes training on different cognitive domains such as orientation, attention, memory, executive functions, visuospatial skills, language, and social cognition while providing scientific education on each one. It also offers compensatory strategies to address and recognize features of healthy aging, such as benign forgetfulness and forgetfulness that are mostly related to dementia. This model consists of ten sessions of 45 minutes each. In each session, cognitive stimulation is performed, and material to work on at home is provided. Throughout these ten sessions, topics concerning psychoeducation are addressed, and common objectives are established with the patient. The program tackles five protective factors for cognitive impairment (cognitive stimulation, nutrition, physical exercise, social activities, and control of cardiovascular risk factors) that favor healthy aging. The program also provides external strategies that will enable the patient to compensate for memory difficulties. Other topics addressed by the program include the concept of neuroplasticity, the importance of remaining active during later life, the association between attention and memory, and how to become an active observer (Table 1).

**Table 1:** Content of the rehabilitation sessions.

Session 1	Introduction to cognitive rehabilitation. Agreeing on goals. Psychoeducation on how to maintain an active brain. Development of five factors for staying healthy: physical activity, cognitive stimulation, social activities, healthy eating and control of cardiovascular risk factors. Concepts of neuroplasticity and cognitive reserve.
Session 2	Importance of information coding. How to optimize the recording of data entering my mind. Cognitive exercise and homework to implement amnesic or organizational compensation strategies, as appropriate.
Session 3	Attention; presentation of the different types. Keys and tips for better functioning and impact on memory. Cognitive exercise and homework to implement amnesic or organizational compensation strategies, as appropriate.
Session 4	Memory; processes and phases. Organizational strategies, information grouping and external aids. Cognitive exercise and homework to implement amnesic or organizational compensation strategies, as appropriate.
Session 5	Memory strategies. The importance of association and visualization. Cognitive exercise and homework to implement amnesic or organizational compensation strategies, as appropriate.
Session 6	Information on how mood, sleep and context impact on memory. Techniques to identify automatic thoughts that influence distracting phase one memory. Cognitive exercise and homework to implement amnesic or organizational compensation strategies, as appropriate.
Session 7	Incorporation of internal strategies to improve information encoding. Techniques to improve mood and stress. Mindfulness. Cognitive exercise and homework to implement amnesic or organizational compensation strategies, as appropriate.
Session 8	Executive Functions; What are they for? How do they influence my routine? Exercising information processing speed. Cognitive exercise and homework to implement amnesic or organizational compensation strategies, as appropriate.
Session 9	Integration of the given strategies for an optimization of attention and memory in the daily routine. Review of these strategies. Cognitive exercise and homework in order to implement amnesic or organizational compensation strategies, as appropriate.
Session 10	Review of the material and closing. “Memory is context-dependent, you can’t talk about how it works on its own”. Cognitive exercise and homework to implement amnesic or organizational compensation strategies, as appropriate.

Participants were evaluated pre-treatment (week 0) and post-treatment (week 10). Likewise, informants, patients’ caregivers or relatives answered questionnaires about the patient’s symptoms and their autonomy for activities of daily living. For the analysis of the data, the intention-to-treat criterion was used.

### Cognitive, neuropsychiatric and functional assessment

Cognitive screening was performed using the Argentine version of the Montreal Cognitive Assessment (MoCA)^20^.

The pre- and post-treatment cognitive assessment consisted of: the Rey Auditory Verbal Learning Test (RAVLT)^21^, the Verbal Fluency test^22^, the Geriatric Depression Scale (GDS)^23^, the Neuropsychiatric Inventory Questionnaire (NPI-Q)^24^, the Functional Activities Questionnaire (FAQ)^25^, the Oblivion Detection Scale (EDO-10)^26^ and the Multifactorial Memory Questionnaires (MMQ)^27^, and Depression, Anxiety and Stress Scale (DASS-21)^28^. Among these scales, those directed to the patient evaluate cognitive aspects such as verbal memory (RAVLT), language (verbal fluency), subjective satisfaction with memory, implementation and acquisition of amnestic strategies (MMQ), psychiatric symptomatology such as symptoms of depression (GDS), anxiety and stress (DASS-21). Moreover, the scales completed by the informant refer to symptoms such as delirium, apathy, irritability, changes in eating or sleeping habits (NPI-Q); cognitive symptoms such as forgetfulness, executive failures, distractions, anosognosia (EDO-10), and functional capacity in basic activities of daily living (FAQ).

### Statistical analysis

All variables were tested graphically and analytically for normality assumptions. Summary statistics are presented as mean and standard deviation (SD) for variables with normality assumptions. When appropriate, categorical and normally distributed variables were analyzed through Pearson’s chi-square test and Student’s *t*-test. Non-normally distributed variables were analyzed with Mann-Whitney U test.

Data analysis was implemented in R, 4.0.5 (R Foundation) using hierarchical mixed-effects models. Group (control/treatment) and time (pre/post-intervention) were treated as fixed factors and participants’ ID as a random effect. We analyzed changes (pre/post-intervention) in each measure of the cognitive assessment (MoCA, RAVLT, GDS, Verbal Fluency, NPI-Q, EDO-10, MMQ, DASS-21), and controlling for diagnosis, age and sex.

## RESULTS

Before treatment, the groups did not differ in the MoCA score (p=0.22), nor did they differ in age (p=0.63) or sex (p=0.70) (Table 2 and 3).

**Table 2:** Demographic results.

Characteristic		Control (n=30)*	Treatment (n=30)*	p-value†
Age (years)		72.700 (5.150)	71.100 (8.431)	0.639
Sex	Male (%)	24 (40)	30 (50)	0.700
	Female (%)	36 (60)	30 (50)	

Notes:

* Controls refers to patients on the waiting list who have not received cognitive rehabilitation to date. Treatments refers to patients who have received CTR

† In the present study, a p-value of less than 0.05 is considered statistically significant.

**Table 3:** Pre-treatment results.

Test	Control (n=30)*	Treatment (n=30)*	p-value
MoCA	-2.970 (2.913)	-1.969 (2.504)	0.220
RAVLT total	-3.204 (1.758)	-2.032 (1.607)	0.067
RAVLT delayed recall	-2.583 (1.048)	-1807 (1.332)	0.086
Recognition	-2.380 (1.941)	-1.779 (2.254)	0.656
Falses positives	2.933 (2.947)	2.267 (2.876)	0.840
Instrusions	2.800 (2.734)	2.833 (2.627)	0.840
Semantic fluency animals	-1.355 (1.096)	-1.425 (.1142)	0.395
Semantic fluency vegetables	-1.175 (0.836)	-1.103 (0.908)	0.721
Phonological fluency	-1.479 (1.267)	-1.817 (1.145)	0.130
GDS	3.167 (3.281)	3.733 (2.778)	0.675
FAQ	7.767 (9.008)	5.883 (7.259)	0.746
NPIQ	5.933 (6.638)	4.533 (5.643)	0.384
EDO–10	4.667 (3.477)	3.900 (3.089)	0.846

Abbreviations: MoCA: Montreal Cognitive Assessment; RAVLT: Rey Auditory Verbal Test; GDS: Geriatric Depression Scale; FAQ: Functional Activities Questionnaire; NPI-Q: Neuropsychiatric Inventory Questionnaire; EDO-10: Oblivion Detection Scale.

Note:

* Data are reported as mean Z (standard deviation error).

**Figure 1:**
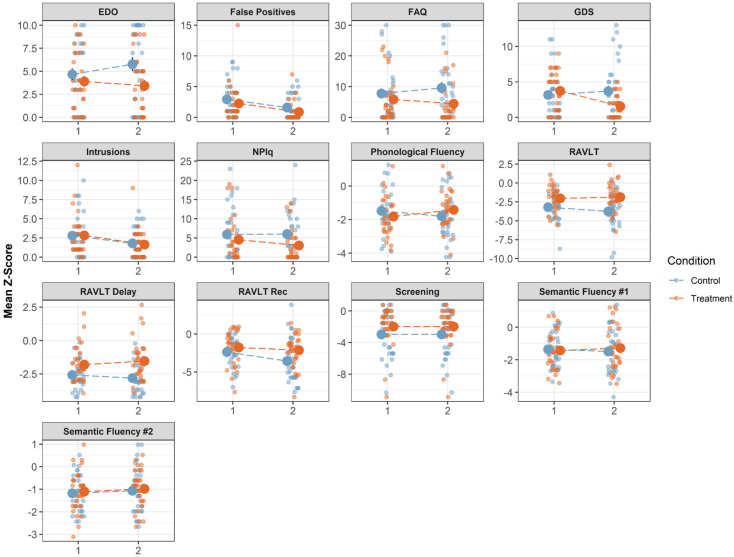
Cognitive performance in two timepoints for treatment and control group.

**Figure 2:**
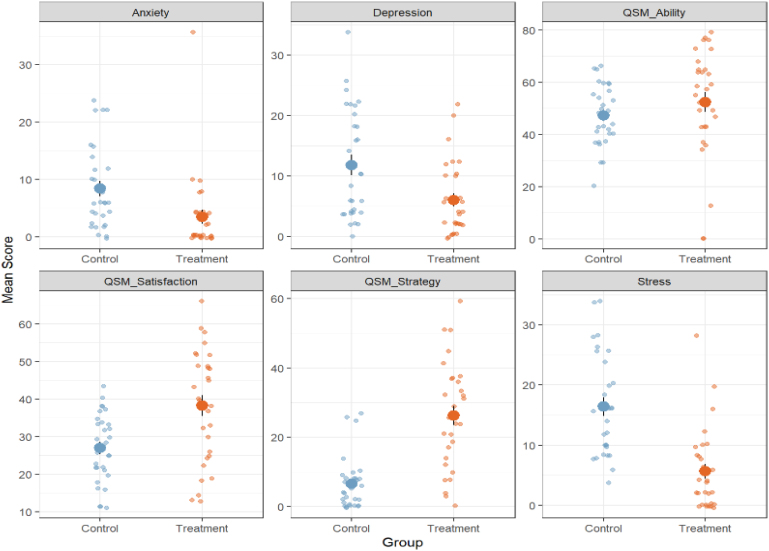
Depression, Anxiety and Stress Scale subscores and subjective memory subscores in two time points for treatment and control groups.

Mixed model results showed for each measure of interest a significant group (control/treatment) x time (pre/post) interaction, revealing that the treatment group at week 10 had better scores in cognitive variables (Figures 1 and 2): memory (RAVLT learning trials β=0.7; p=0.030); RAVLT delayed recall (β=0.48; p=0.029), phonological fluency (β=0.72; p=0.001), activities of daily living (FAQ β=-3.16; p=0.001), satisfaction with memory performance (MMQ satisfaction β=10.3; p=0.004) and use of memory strategies (MMQ strategy β=4.4; p=0.00), as well as a significant reduction of affective symptomatology: depression (GDS β=-2.68; p=0.00), neuropsychiatric symptoms (NPI-Q β=-1.46; p=0.045), forgetfulness (EDO-10 β=-1.5; p=0.00), and stress (DAS stress β=-6.0; p=0.00). Thus, the provided treatment would appear to be beneficial for cognition, psychiatric symptoms and daily life functioning for patients with MCI (Tables 4 and 5).

**Table 4:** Results post-treatment.

Test	Control (n=30)*	Treatment (n=30)*	p-value
MoCA	-2.970 (2.913)	-1.969 (2.504)	0.220
RAVLT total	-3.750 (2.299)	-1.867 (1.887)	0.030
RAVLT delayed recall	-2.815 (1.189)	-1.533 (1.539)	0.029
Recognition	-3.555 (2.951)	-2.130 (2.110)	0.206
Falses positives	1.600 (1.993)	0.867 (1.613)	0.862
Instrusions	1.800 (2.041)	1.633 (1.866)	0.862
Semantic fluency animals	-1.486 (1.390)	-1.279 (1.349)	0.218
Semantic fluency vegetables	-1.066 (1.038)	-0.984 (0.830)	0.925
Phonological fluency	-1.803 (1.396)	-1.414 (1.115)	0.001
GDS	3.700 (4.044)	1.567 (1.547)	0.000
FAQ	9.600 (9.754)	4.467 (5.998)	0.001
NPIQ	6.033 (6.049)	3.067 (4.085)	0.045
EDO–10	5.767 (4.014)	3.433 (3.224)	0.000

Abbreviations: MoCA: Montreal Cognitive Assessment; RAVLT: Rey Auditory Verbal Test; GDS: Geriatric Depression Scale; FAQ: Functional Activities Questionnaire; NPI-Q: Neuropsychiatric Inventory Questionnaire; EDO-10: Oblivion Detection Scale.

Note:

* Data are reported as mean z (standard deviation error).

**Table 5:** Results Depression, Anxiety and Stress Scale and Multifactorial Memory Questionnaires.

Test	Control (n=30)*	Treatment (n=30)*	p-value
DASS–21 anxiety	8.700 (7.190)	3.467 (6.907)	0.110
DASS–21 depression	11.800 (9.133)	6.000 (6.011)	0.829
DASS–21 stress	16.400 (8.459)	5.667 (6.666)	0.000
MMQ ability	47.233 (11.649)	52.300 (20.785)	0.348
MMQ satisfaction	26.933 (9.082)	38.200 (15.048)	0.004
MMQ strategy	6.600 (7.623)	26.300 (15.182)	0.000
DASS 21 anxiety	8.400 (7.190)	3.467 (6.907)	0.110
DASS 21 depression	11.800 (9.133)	6.000 (6.011)	0.829
DASS 21 stress	16.400 (8.459)	5.667 (6.666)	0.000
MMQ ability	47.233 (11.649)	52.300 (20.785)	0.348
MMQ satisfaction	26.933 (9.082)	38.200 (15.048)	0.004

Abbreviations: DASS-21: Depression, Anxiety and Stress Scale; MMQ: Multifactorial Memory Questionnaires.

Note:

* Data are reported as mean Z (standard deviation error).

## DISCUSSION

We studied patients diagnosed with MCI who attended a neurological clinic for CTR. Unlike the control group, our results showed that treatment patients benefited in multiple cognitive domains: verbal learning, phonological fluency, subjective memory satisfaction, implementation of music strategies, neuropsychiatric symptoms, and mood.

Regarding cognitive benefits, our results were consistent with other studies that presented a significant increase in cognitive benefits, both for mild cognitive impairment and brain injuries^29–32^, exposing improvements in instrumental activities of daily living, in neuropsychiatric symptoms, and mood. However, it should be emphasized that the results in this regard are currently scarce and that the methodology used is widely variable due to difficulties in obtaining homogeneity in treatment and patient groups.

Our study is one of the first in our region to assess the effects of CTR showing an effective intervention to improve performance in cognitive variables and reduce neuropsychiatric symptomatology compared to untreated patients with MCI. These results have great significance in the context of the COVID-19 pandemic in South America.

Likewise, these results are an initial step in our attempts to establish a framework and promote Latin American clinical research on CTR, which we believe will help provide more culturally appropriate guidelines unique to the region. As a result of the uniqueness and heterogeneity of the region, we urge local organizations to work with CTR in order to enhance the quality of treatments.

The study of neuropsychology must swiftly change in response to public health concerns and social distancing instructions in reaction to the COVID-19 pandemic. Enough evidence justifies the use of video conferencing technology for remotely delivering neuropsychological treatment. CTR can be provided to patients who have cognitive impairment, but not to those with visual or auditory impairments, history of severe major neurocognitive disorder, acute confusional episodes, or substantial communication issues^33^.

While protecting the vulnerable population from COVID-19 infection, social isolation measures can also hold the elderly population at risk, affecting the continuity of medical treatment, including CTR. In many cases, these measures have caused MCI patients to abandon their face-to-face CR treatments, generating a future cost and burden for the State, families, and individuals^17^. This study focused on the cognitive and neuropsychiatric effects of CTR on MCI patients compared to a control group. Our results highlight the importance of performing CTR treatment in patients with MCI.

As a final point, our study has some limitations:

- Heterogeneity of MCI subtypes,
- Small sample size, and
- CTR group was compared with a group of untreated patients. Future studies should include a systematic analysis with larger patient cohorts and measurement of comparative effects between conventional CTR.

Likewise, considering the sociocultural heterogeneity in Latin America, it would be interesting to replicate the study in other countries of our region.
